# Risk of cardiac arrhythmias after electrical accident: a single-center study of 480 patients

**DOI:** 10.1007/s00392-019-01420-2

**Published:** 2019-02-15

**Authors:** David Pilecky, Mate Vamos, Peter Bogyi, Balazs Muk, Dora Stauder, Hajnalka Racz, Noemi Nyolczas, Gabor Z. Duray, Gabor Zacher, Endre Zima

**Affiliations:** 1 Department of Internal Medicine III, Klinikum Passau, Innstrasse 76, 94032 Passau, Germany; 2 Department of Cardiology, University Hospital Frankfurt, Goethe University, Theodor-Stern Kai 7, 60590 Frankfurt, Germany; 3 Department of Cardiology, Medical Centre, Hungarian Defence Forces, Róbert K. krt. 44, Budapest, 1134 Hungary; 4 Department of Emergency Medicine, Medical Centre, Hungarian Defence Forces, Róbert K. krt. 44, Budapest, 1134 Hungary; 5 Semmelweis University Heart and Vascular Centre, Városmajor u. 68, Budapest, 1122 Hungary

**Keywords:** Electrical accident, Arrhythmia, Cardiac monitoring, Cardiac necroenzymes

## Abstract

**Objective:**

Patients with electrical injury are considered to be at high risk of cardiac arrhythmias. Due to the small number of studies, there is no widely accepted guideline regarding the risk assessment and management of arrhythmic complications after electrical accident (EA). Our retrospective observational study was designed to determine the prevalence of ECG abnormalities and cardiac arrhythmias after EA, to evaluate the predictive value of cardiac biomarkers for this condition and to assess in-hospital and 30-day mortality.

**Methods:**

Consecutive patients presenting after EA at the emergency department of our institution between 2011 and 2016 were involved in the current analysis. ECG abnormalities and arrhythmias were analyzed at admission and during ECG monitoring. Levels of cardiac troponin I, CK and CK-MB were also collected. In-hospital and 30-day mortality data were obtained from hospital records and from the national insurance database.

**Results:**

Of the 480 patients included, 184 (38.3%) had suffered a workplace accident. The majority of patients (96.2%) had incurred a low-voltage injury (< 1000 V). One hundred and four (21.7%) patients had a transthoracic electrical injury while 13 (2.7%) patients reported loss of consciousness. The most frequent ECG disorders at admission were sinus bradycardia (< 60 bpm, *n* = 50, 10.4%) and sinus tachycardia (> 100 bpm, *n* = 21, 4.4%). Other detected arrhythmias were as follows: newly diagnosed atrial fibrillation (*n* = 1); frequent multifocal atrial premature complexes (*n* = 1); sinus arrest with atrial escape rhythm (*n* = 2); ventricular fibrillation terminated out of hospital (*n* = 1); ventricular bigeminy (*n* = 1); and repetitive nonsustained ventricular tachycardia (*n* = 1). ECG monitoring was performed in 182 (37.9%) patients for 12.7 ± 7.1 h at the ED. Except for one case with regular supraventricular tachycardia terminated via vagal maneuver and one other case with paroxysmal atrial fibrillation, no clinically relevant arrhythmias were detected during the ECG monitoring. Cardiac troponin I was measured in 354 (73.8%) cases at 4.6 ± 4.3 h after the EA and was significantly elevated only in one resuscitated patient. CK elevation was frequent, but CK-MB was under 5% in all patients. Both in-hospital and 30-day mortality were 0%.

**Conclusions:**

Most of cardiac arrhythmias in patients presenting after EA can be diagnosed by an ECG on admission, thus routine ECG monitoring appears to be unnecessary. In our patient cohort cardiac troponin I and CK-MB were not useful in risk assessment after EA. Late-onset malignant arrhythmias were not observed.

## Introduction

Electrical accidents (EA) are rare, but can cause serious and potentially life-threatening injuries to multiple organs. The majority of epidemiological data refers to workplace accidents which account for a significant share of such accidents in adults, although large regional variability exists between European countries in terms of incidence and mortality [1]. Generally favourable outcomes are reported. For instance, from the 3463 workplace-related EAs reported in 2016 in Germany, there were only five fatalities [2].

The severity of electrical burns and injuries to internal organs depends on voltage, resistance of the body, duration of current flow, type of current (direct or alternating) and the path of current through the body. Transthoracic current may lead to cardiac complications which manifest predominantly as arrhythmias, conduction disturbances, and myocardial tissue damage, depending mainly on the strength of current [3].

Arrhythmias resulting from the proarrhythmic effect of electric shock usually occur immediately after the accident. If electric current reaches the heart within the vulnerable period it may also cause ventricular fibrillation (VF), which is the most common cause of death after EA [4]. In patients presenting at emergency units after EA, the most commonly diagnosed arrhythmias are sinus tachycardia, sinus bradycardia and isolated premature atrial and ventricular complexes (PACs and PVCs) [5–7]. If the conduction system of the heart is affected, bundle branch block, and various degrees of atrioventricular block may also occur [6, 8, 9], however, the exact frequency of these arrhythmias is unknown. Late-onset malignant arrhythmias are very rare after EA. Only a few case reports have described delayed malignant arrhythmias, and only two of these cases have been documented with an initial ECG [9–11].

According to the current guidelines of the European Resuscitation Council (ERC), ECG monitoring is recommended after EA for patients with known cardiorespiratory disease or one or more of the following risk factors: loss of consciousness, initial cardiac arrest, soft tissue damage and burns, or ECG-abnormalities at the time of admission [12]. Notably, these guideline recommendations are based only on a few, mostly retrospective studies and case reports with low evidence level.

We sought to analyze the data of consecutive patients who presented at the emergency unit after EA to determine the frequency of ECG abnormalities and cardiac arrhythmias, and to evaluate the predictive value of some parameters that characterize patients and EA. Furthermore, we assessed the risk of late-onset malignant arrhythmias, and in-hospital and 30-day mortality.

## Methods

### Patient population

Consecutive patients who were admitted with EA (ICD diagnosis T75.4, effects of electric current) to the Emergency Department of the Medical Centre, Hungarian Defence Forces (Budapest, Hungary) between 01.01.2011 and 31.12.2016 were involved in the current analysis. This department is the largest multidisciplinary emergency center in Budapest (admissions in 2016: 47,734) and is prepared for all types of major adult emergencies, including burn victims.

Clinical data were obtained from the hospital information system and patient records. Baseline demographics, medical history, and antiarrhythmic medication were registered along with location, time and circumstances of the EA. Transthoracic current was defined similarly to the study of Bailey et al. based on sensation of the patient, burns marks or accident mechanism [7]. Furthermore, all clinical parameters which are deemed to be risk factors for cardiac arrhythmias based on the ERC criteria were summarized. We also recorded presenting symptoms, severity of burns, and other injuries.

### Biochemical and electrocardiographic analysis

The following laboratory parameters were identified: serum sodium, potassium and creatinine, high sensitive cardiac troponin I (cTnI), creatine kinase (CK), and creatine kinase muscle–brain ratio (CK-MB%). The upper limit of the normal level of these parameters is defined at our institution as 0.04 ng/mL for cTnI, 190 U/mL for CK and 5% for CK-MB%.

Pre-hospital and in-hospital ECGs were blind analyzed by two independent cardiologists, who were not aware of the clinical presentation or the purpose of the study. In the case of disagreement, a third expert was involved. Length of ECG monitoring, arrhythmic events during ECG monitoring, length of stay at the emergency department (ED) and the disposition decisions were also reviewed.

### Survival analysis

Data for all-cause and arrhythmic cause in-hospital and 30-day mortality were collected. Mortality data were obtained from the hospital records and from the up-to-date database of the National Health Insurance Fund of Hungary. Registration at this site enables the treating physician to check whether the unique health insurance number of his/her patient is active or deactivated. All those patients who died are set immediately to “passive” concerning the insurance state of them.

### Statistical analysis

All relevant patient data were recorded in an anonymized form in a Microsoft Excel 2007 spreadsheet (Microsoft, Redmont, WA). For statistical analysis we used the statistical program R (The R Foundation for Statistical Computing, version 3.5.0). Descriptive statistics for categorical variables are shown as percentages, while continuous variables are represented by their means and standard deviations. Multivariable logistic regression was used to determine independent risk factors for the occurrence of arrhythmias. The clinical parameters analyzed in this model were structural heart disease, loss of consciousness, high voltage electric shock, transthoracic current, burns and soft tissue injuries.

This non-interventional observational retrospective study was undertaken in conformity with the Helsinki Declaration. Concerning the retrospective data collecting and analyzing manner of the study, the authors have applied for and achieved the approval of the Regional Ethics Committee without the obligatory need of written consent of the subjects involved in the analysis based on the regulations of the Semmelweis University.

## Results

### Patient population

**Fig. 1:**
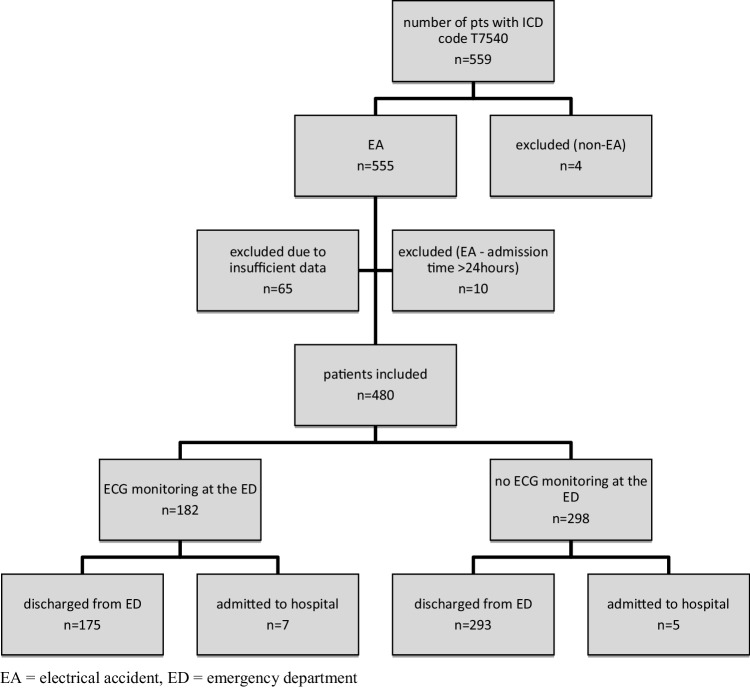
Patient selection and disposition of patients included in the study. *EA* Electrical accident, *ED* emergency department

During the study period, 559 patients were admitted to the ED with a first diagnosis of EA. Seventy-nine patients were excluded for various reasons which are summarized in Fig. 1. The final analysis assessed 480 patients (287 males) with a mean age of 34.3 years (Table 1). Workplace accidents accounted for 38.3% of cases; in the majority of these work-related accidents men were affected (72%). More than 60% of patients had no complaints on admission, while the most common complaints were as follows: numbness of extremities (19.6%), burns (17.5%), and chest pain (5.8%). Circumstances of EA are detailed in Table 2. Small electricity-related marks or mild first-degree burns were found in 75 patients (15.6%). Eight patients had second-degree, one patient third-degree, and two patients fourth-degree burn injuries. Treatment by a burn care specialist was needed in eight cases (Table 2). In further eight patients concomitant traumatic injuries were observed, such as contusions, fractures, or ceratoconjunctivitis photoelectrica.

**Table 1:** Demographic characteristics and history of final patient sample

Number of patients (male)	480 (287)
Age (years; mean ± SD)	34.3 ± 11.6
History	*n* (%)
Hypertension	45 (9.4%)
Asthma or COPD	10 (2.1%)
Structural heart disease	7 (1.5%)
Atrial fibrillation	2 (0.4%)
Actual pregnancy	6 (1.3%)
Medication	
Beta-blockers	22 (4.6%)
Other antiarrhythmic agent	0 (0%)

*SD* Standard deviation

**Table 2:** Presentation and circumstances of electrical accident

Baseline parameters	
Self-admission	231 (48.1%)
Admission via ambulance car	245 (51.0%)
Admission via emergency helicopter	4 (0.8%)
Time from accident to admission (h; mean ± SD)	2.9 ± 3.9
Resuscitation on site	1 (0.2%)
High voltage accident (> 1000 V)	18 (3.8%)
Loss of consciousness	13 (2.7%)
Transthoracic current	104 (21.7%)
Workplace accident	184 (38.3%)
Suicide attempt	5 (1.0%)
Power source	
Household electrical device	129 (26.9%)
Electric conductor	79 (16.5%)
Electric socket	69 (14.4%)
Lamp	44 (9.2%)
Other	61 (12.7%)
Not known	98 (20.4%)
Complaints at admission	
No complaints	298 (62.1%)
Numbness of extremities	94 (19.6%)
Burns	84 (17.5%)
Chest pain	28 (5.8%)
Pain of the extremities	21 (4.4%)
Headache	14 (2.9%)
Dizziness	13 (2.7%)
Palpitations	9 (1.9%)
Nausea	6 (1.3%)
Intubated	4 (0.8%)
Other	14 (2.9%)
Severity of burns	
I° or electrical marks	75 (15.6%)
II°	8 (1.7%)
III°	1 (0.2%)
IV°	2 (0.4%)
Concomitant injuries	8 (1.7%)

### Arrhythmias at admission

Pre-hospital ECG was performed on 205 patients (42.7%). ECG on admission was available for almost all patients (*n* = 475); however, pre-discharge ECG was performed only in 76 cases (Table 3).

**Table 3:** Arrhythmias and further electrocardiographic changes

Prehospital ECG available	205 (42.7%)
ECG on admission available	475 (99.0%)
Control ECG before discharge available	76 (15.8%)
Arrhythmias	
Sinus bradycardia (< 60 bpm)	50 (10.4%)
Sinus tachycardia (> 100 bpm)	21 (4.4%)
Sinus arrest with atrial escape rhythm	2 (0.4%)
Newly diagnosed atrial fibrillation	1 (0.2%)
Frequent multifocal atrial premature complexes	1 (0.2%)
Regular SVT during monitoring	1 (0.2%)
Atrial fibrillation during monitoring	1 (0.2%)
Ventricular fibrillation before admission	1 (0.2%)
Non-sustained ventricular tachycardia	1 (0.2%)
Ventricular bigeminy	1 (0.2%)
Further electrocardiographic observations	
Non-specific ST-T changes	41 (8.5%)
Incomplete RBBB	19 (4.0%)
PQ depression	9 (1.9%)
Non-specific IVCD	6 (1.3%)
Single PVC	5 (1.0%)
RBBB (QRS duration ≥ 0.12 s)	4 (0.8%)
Left anterior hemiblock	3 (0.6%)
Single PAC	2 (0.4%)
First-degree AV block	1 (0.2%)
Trifascicular block	1 (0.2%)
Ventricular preexcitation syndrome	1 (0.2%)

*SVT* Supraventricular tachycardia, *RBBB* right bundle branch block, *AV* atrioventricular, *IVCD* intraventricular conduction delay, *PVC* premature ventricular complex, *PAC* premature atrial complex

The most frequent supraventricular arrhythmias on admission were mild sinus bradycardia (< 60 bpm, *n* = 50, 10.4%) and sinus tachycardia (> 100 bpm, *n* = 21, 4.1%). All patients with sinus bradycardia were asymptomatic and did not require any intervention. Atrial fibrillation (AF) was detected in two cases on the admission ECG. One of these patients had AF as a first diagnosis. A few hours later, this patient had a spontaneous conversion to sinus rhythm. In one young patient, frequent multifocal PACs were observed on the pre-hospital and admission ECGs which spontaneously resolved during monitoring. In two other patients intermittent sinus arrest with atrial escape rhythm without haemodynamic instability was observed.

The detected ventricular arrhythmias were as follows: one male patient was resuscitated due to VF at the site of the accident. On admission this patient had sinus tachycardia but no malignant arrhythmias occurred during further monitoring in the ICU. In another patient, ventricular bigeminia was found on the admission ECG, which became less frequent during monitoring, and only single PVCs were seen on the control ECG before discharge. This patient did not have a known history of PVC. In another patient with a history of severe alcohol abuse but without known heart disease recurrent non-sustained ventricular tachycardia (nsVT) was observed. Electrolyte disorder and myocardial necrosis were excluded, while a transthoracic echocardiography did not show significant abnormalities. After administration of a single dose of amiodarone (150 mg iv.), nsVTs were terminated. The patient was monitored for 18 h and then left the hospital against medical advice.

Further ECG abnormalities observed on admission are summarized in Table 3. The most common ECG changes were non-specific ST-T abnormalities (e.g. early repolarization) and incomplete right bundle branch block. We also compared ECG curves in cases where more than one ECG was available (*n* = 246). In 12 cases intermittent incomplete right bundle branch block was detected. Except for the cases reported here, we found no further dynamic ECG changes.

### Arrhythmias during ECG monitoring

Asyptomatic patients without risk factors and without ECG abnormalities were discharged directly from the ED. ECG monitoring was performed in 182 (37.9%) patients for 12.7 ± 7.1 h at the observation unit of the ED. In one patient, a symptomatic regular supraventricular tachycardia was detected, that was terminated via vagal maneuver, although this patient suffered from recurrent palpitations even before the EA. In another patient with known paroxysmal AF, an AF episode was documented. Up to this two cases no further clinically relevant arrhythmias were detected.

Multivariable logistic regression indicated no statistically significant association between the most important baseline clinical parameters and the occurrence of the arrhythmias listed in Table 3; however, for high-voltage injury borderline significance was detected (Table 4). Similar results were found when patients presenting with sinus tachycardia or sinus bradycardia were excluded from the regression model.

**Table 4:** Association between various clinical parameters and arrhythmias

Variable	OR (95% confidence interval)	*p* value
Structural heart disease	0.90 (0.11–7.60)	0.92
LOC	2.82 (0.75–10.60)	0.13
High voltage	2.94 (0.91–9.53)	0.07
Transthoracic current	1.49 (0.85–2.63)	0.13
Burns and soft tissue injuries	0.14 (0.01–1.55)	0.11

*OR* Odds ratio, *LOC* loss of consciousness

### Biochemical analysis

Results of laboratory tests are presented in Table 5. High-sensitive cTnI and CK was available in 354 (73.8%) patients performed at an average of 4.6 (± 4.3) h after the EA. In most patients (*n* = 347) cTnI was below the upper limit of normal (< 0.04 ng/mL). Slightly elevated cTnI (0.04–0.4 ng/mL) was found in six patients, while one patient had moderate cTnI elevation at 5.40 ng/mL. This patient was resuscitated by the emergency service on site due to ventricular fibrillation. Patients with mild cTnI elevation showed no ECG abnormalities. CK elevation (> 170 U/L) was detected in 120 patients and may be explained by soft tissue injuries, burns, muscle pain, or transthoracic current in 74 patients. The CK-MB ratio was < 5% in all patients. All patients had a normal renal function and electrolyte levels at admission.

**Table 5:** Laboratory findings

Laboratory values available	354 (73.8%)
Time from EA to blood sample (h)	4.6 ± 4.3
Serum sodium (mmol/L)	141 ± 2
Serum potassium (mmol/L)	3.9 ± 0.4
Serum creatinine (mg/dL)^a^	0.9 ± 0.2
cTnI slightly elevated (0.04–0.4 ng/mL)	6 (1.25%)
cTnI significantly elevated (> 0.4 ng/mL)	1 (0.2%)
CK (U/L)	305 ± 1356
CK elevated (> 170U/L)	120 (25.0%)
CK-MB%^b^	1.1 ± 0.6
CK-MB% elevated (> 5%)	0 (0%)

Values are reported as percentile and mean ± standard deviation

*EA* Electrical accident, *cTnI* cardiac troponin I, *CK* creatine kinase, *CK-MB* creatine kinase muscle and brain

^a^The estimated glomerular filtration rate (eGFR) was above 60 mL/min in each patient

^b^CK-MB% values were available for 290 (60.6%) patients

### In-hospital and post-discharge follow-up

The overwhelming majority of patients (*n* = 468; 97.5%) were discharged from the ED, while only 12 patients were admitted for further observation or treatment. Among them, four patients were admitted to the multidisciplinary intensive care unit, one to the burn unit, four to the psychiatric ward, two to the cardiology ward and one to the general internal medicine ward. All hospitalized patients were discharged from hospital to home. A 30-day follow-up was undertaken for 477 patients, while three patients were lost to follow-up. At the end of this period, all patients had an active insurance status and was assumed to be alive (Table 6).

**Table 6:** Disposition of patients and survival

ED length of stay (h; mean ± SD)	6.9 ± 5.7
ECG monitoring at ED	182 (37.9%)
Duration of ECG monitoring at ED (h; mean ± SD)	12.7 ± 7.1
Admitted for further observation/treatment	12 (2.5%)
Patients discharged from hospital	480 (100%)
Follow-up completed	477 (99.4%)
30-day survival	477 (100%)

*ED* Emergency department, *SD* standard deviation

## Discussion

### Main findings

To the best of our knowledge, the present analysis is the largest study thus far published to focus on arrhythmias and cardiac biomarker changes following EA. We found that all arrhythmias with possible relation to EA in patients presenting after EA could be diagnosed by ECG on admission. Late-onset malignant arrhythmias were not observed at all and the in-hospital and 30-day mortality were 0%. In our patient cohort, elevation of cTnI was rare and was not associated with arrhythmias. CK-MB% was also not useful in risk assessment after EA.

### Arrhythmias after EA

Arrhythmias caused by electric shock usually occur immediately after EA, and can directly lead to death [13]. A few cases of late-onset malignant arrhythmias after EA have been reported, but only two of them were documented with an initial ECG. In the first case, published by Sharma et al., a progressive AV-block was detected after an electric shock of 220–240 V, followed by ventricular fibrillation a few hours later [9]. The second case report describes a patient who developed pulseless ventricular tachycardia within 24 h after hospitalization which was terminated with DC-shock. The admission ECG showed a prolonged QTc interval (500 ms) while marked fragmentation of the QRS complex was also observed. At 1 month’s follow-up these ECG abnormalities were normalized [10].

Some previous reports have investigated the risk of arrhythmias after EA in a systematic fashion. Pawlik et al. retrospectively investigated 240 patients who suffered electric shock and were admitted to an ED. 62% of patients were monitored for an average of 4.25 h, during which time no malignant arrhythmias occurred. 90-day mortality was 0% for all patients [14]. Similar results were found in the retrospective analysis of Searle et al. All 262 patients involved in the study were monitored for more than 12 h: no life-threatening arrhythmias were observed and in-hospital mortality was 0% [6]. The prospective multicentre study published by Bailey et al. involved only patients (*n* = 134) with one or more risk factors according to the current ERC guideline. Malignant arrhythmias did not occur in any of the patients during a 24-h period of monitoring and there were no late cardiac complications during the 1 year follow-up [7].

A recent, nationwide, Danish register-based study reviewed 11,462 patients who presented at the emergency ward or were admitted to hospital after EA. The occurrence of documented cardiac procedures was very low during the 1-year follow-up period and in no case could a relationship between the cardiac event and electric accident be identified. The 5-year mortality of EA survivors was similar to that of the matched patient population, regardless of whether the patient was admitted to hospital or discharged directly from ED [15].

The results of our high-volume retrospective analysis confirm the results of the previous studies. Clinically relevant arrhythmias were rare in patients presenting after EA at the ED and could be diagnosed based on admission ECG. No new-onset arrhythmias were observed in patients who underwent cardiac monitoring, except for two cases where regular supraventricular tachycardia and an AF episode was detected. Some of the detected arrhythmias may be explained as physiological responses or normal variability such as sinus tachycardia due to pain or anxiety, or sinus bradycardia in young and physically fit patients. Other of the observed ECGs could be classified as borderline changes that should not necessarily be considered as pathological findings (e.g. non-specific ST changes or incomplete RBBB).

### Predictive value of biomarkers after EA

There is insufficient evidence about the role of cardiac biomarkers in risk stratification after EA. A small prospective study found higher N-terminal pro b-type natriuretic peptide levels in patients with high-voltage electric injury and arrhythmia [16]. In the same study, CK-MB and cTnI were not found to be higher in arrhythmic patients compared to patients without arrhythmia after EA. Several other studies also suggest that CK-MB is not a reliable marker for screening arrhythmic and cardiac complications as this can be also elevated due to skeletal muscle and soft tissue damage [17, 18]. Although cTnI is a much more sensitive cardiac biomarker than CK or CK-MB, it does not usually increase after an EA. Troponin elevation is only observed in some rare cases, is usually without clinical relevance, and no data support the claim that cTnI elevation can predict arrhythmias after EA [6, 7, 14]. The arrhythmogenic effects of electric shocks are not considered to be primarily due to myocardial necrosis. This hypothesis is supported by the histopathological observation that the most common change in victims of electrocution is myofibre break-up leading to inhomogenity of the conduction system of the heart [19]. Rarely transient Brugada type repolarisation abnormalities may occur after AE caused possibly through an imbalance of ion currents leading to an arrhythmogenic trigger [20–22].

Although CK elevation was relatively common in our patient cohort, CK-MB% was below 5% in each case. It is known, that electrical injury can lead to rhabdomyolysis which is related to potential risk of acute kidney injury [23]. In our analysis some patients with high-voltage accident and/or severe burn injuries showed a massive elevation of CK level suggesting rhabdomyolysis, however, none of the patients developed an acute renal failure. Significant elevation of cTnI was only detected in one patient who was resuscitated for 25 min due to ventricular fibrillation. TnI elevation is considered to be due to long-term myocardial low perfusion. Sinus tachycardia was seen on the patient’s admission ECG recording without repolarization abnormalities, while control laboratory tests showed no further increase in cTnI levels.

## Conclusion

This analysis of patients suffered predominantly low-voltage electric injury showed that if the patient’s admission ECG is negative, the onset of clinically relevant arrhythmias is still unlikely. As our mortality data suggest, delayed fatal adverse events (e.g. fatal ventricular arrhythmias) did not occur regardless of whether the patient was monitored. Parameters considered to be risk factors such as known structural heart disease, loss of consciousness, high voltage electric shock, burn and soft tissue injuries were also not significant predictors of the occurrence of arrhythmias. Elevation of cTnI appears to be sporadic and has no predictive value concerning arrhythmias after EA. Measurement of cTnI and CK-MB%, especially in stable patients with no ECG changes, may be unnecessary and may increase costs and patient waiting time.

## Limitations

The main limitation of this work stems from the retrospective nature of data collection. It should also be noted that not all patients were monitored systematically, therefore, non-lethal, and even non-sustained arrhythmias might have occurred while the patients were in the ED but not on a monitor. Furthermore, in a significant proportion of patients no pre-discharge ECG was available. Levels of cardiac necroenzymes were also not available for every patient and there was a considerable variance regarding the time from EA to blood sample. The vaste majority of patients in the sample had suffered a low-voltage electrical injury, so results can not be entirely extrapolated to high-voltage electrical accidents. Finally, we included in our analysis only survivors of EA who presented in the ED. Patients who have not survived an EA and, therefore, have not been referred to hospital for further treatment were not in the focus of this study. However, the number of fatal electrocutions seems to be generally low in Hungary: emergency services were alerted to 847 electrocution cases in 2017, from which seven patients died in the prehospital setting [24].
